# Impact of Atomization Pressure on the Particle Size of Nickel-Based Superalloy Powders by Numerical Simulation

**DOI:** 10.3390/ma15093020

**Published:** 2022-04-21

**Authors:** Yongquan Qing, Kuaikuai Guo, Chen Liu, Youyi Qin, Yu Zhan, Shang Shuo, Yanpeng Wei, Bo Yu, Changsheng Liu

**Affiliations:** 1 School of Materials Science and Engineering, Northeastern University, Shenyang 110819, China; neukuai@163.com (K.G.); liuchenneu@163.com (C.L.); rghsetjrsdkdx@sohu.com (Y.Q.); shangn0000@126.com (S.S.); 2 Key Laboratory for Anisotropy and Texture of Materials, Ministry of Education, Northeastern University, Shenyang 110819, China; 3 State Key Laboratory of Light Alloy Casting Technology for High-End Equipment, Shenyang 110022, China; wyp20@mails.tsinghua.edu.cn (Y.W.); yub@chinasrif.com (B.Y.); 4 School of Science, Northeastern University, Shenyang 110819, China; zhanyu@mail.neu.edu.cn

**Keywords:** computational fluid dynamics, superalloy powders, particle size distribution

## Abstract

Vacuum induction melting gas atomization (VIGA) has evolved as an important production technique of superalloy powders used in additive manufacturing. However, the development of powder preparation techniques is limited because the crushing process of gas-atomized metal melt is difficult to characterize by conventional experimental methods. Herein, we report the application of computational fluid dynamics to simulate the breaking behavior of droplets in the process of preparing nickel-based superalloy powders by VIGA, as well as the results on the effect of gas pressure on the atomization process and powder particle size distribution of metal melt. In the process of primary atomization, the crushing morphology of superalloy melt shows an alternate transformation of umbrella shapes and inverted mushroom cloud shapes, and with the increase in atomization pressure, the disorder of the two-phase flow field increases, which is conducive to sufficient breakage of the melt. Most importantly, in the process of secondary atomization and with the increasing atomization pressure, the particle size distribution becomes narrower, the median particle diameter and average particle size decrease, and the decreasing trend of the particle size increases gradually. The simulation results are compliant with the performed nickel-based superalloy powder preparation tests. This study provides insight into the production and process optimization of superalloy powder prepared by the VIGA method.

## 1. Introduction

With the application of additive manufacturing (3D printing) to produce superalloy or metal parts for aero-engines and gas turbines and for the aerospace industry, the demand for special superalloy or metal powders are gradually increasing [1,2,3,4,5,6]. Currently, vacuum induction melting gas atomization (VIGA) is the most widely used method for preparing superalloy powder. Metal powders prepared by VIGA possess certain advantages, including high purity, good sphericity, low oxygen content, and rapid solidification [7,8,9]. However, superalloy powders prepared by VIGA encounter some problems, such as high cost and unstable properties, which seriously limit the development of 3D printing and powder preparation technology.

The VIGA method produces superalloy powder by impacting and crushing high-temperature metal fluid with high-speed and high-energy inert gas in a very short time [10,11]. This is a complex process of multiphase flow interaction, rapid energy exchange, and numerous parameter variables, including primary and secondary atomization. In the gas atomization process, the chemical composition and superheat of superalloy directly affect the surface tension and viscosity of film melt, so as to affect the particle size of the powder [12,13]. The influence of each control parameter on gas–liquid interaction and droplet breakup still needs to be developed [14]. Conventional experimental methods are inadequate to characterize the entire breakup process, and many complex relationships have not been determined [15,16].

The computational fluid dynamics (CFD) method has attracted increasing attention because it can be applied to complex fluid flow problems, such as the visual reproduction of gas trajectory and metal melt crushing [17,18,19]. In recent years, researchers have used CFD to numerically simulate the gas atomization process. Thompson et al. [20] applied two-way coupling of CFD with a discrete particle model using an Euler–Lagrangian approach to simulate the influence of process parameters on the particle size distribution of metal powder. The authors found that increasing the atomization pressure or the protruding length of the guide pipe leads to a decrease in powder particle size, and increasing the melt surface tension results in an increase in powder particle size. Zhao et al. [21] applied the CFD method to simulate the effect of initial droplet size on the pre-breakup process and found that, with increasing initial droplet size, the TC4 powder particle size gradually increased while the powder particle size distribution became narrower. Shi et al. [22] simulated the atomization process of Fe-based amorphous alloy powder at a gas pressure of 5-8 MPa. The simulation predicted that with the elevation of gas pressure, the particle size decreases gradually, and its distribution becomes narrower, which is consistent with the actual experimental results. Despite notable progress, the effect of high pressure on the atomization process and particle size of superalloy powder has remained elusive due to the different physical properties (such as density, heat capacity, viscosity, surface tension, etc.) between superalloys and other metals [2,23,24].

In this work, we use CFD to simulate the primary and secondary atomization process in the preparation of nickel-based superalloy powder by the VIGA method. The interaction between atomized gas and atomized particles is studied, and the influencing factors and laws of powder particle size and distribution are determined. Furthermore, the outcomes of the simulation are compared with the actual results. Our research aims to provide a theoretical basis for the understanding of atomization and the optimization of process parameters.

## 2. Numerical Simulation Method

In this study, the CFD method in ANSYS Fluent 19.0 commercial software (ANSYS Inc., Canonsburg, PA, USA) is used on an HP Z240 workstation server (Hewlett-Packard, Palo Alto, CA, USA) to simulate the effects of atomization pressure (1–5 MPa) on the gas flow field structure, atomization process, and powder particles during the preparation of nickel-based superalloy powder by the VIGA method. Moreover, argon (99.99%) used in the atomization pulverization process is an ideal gas, and the flow rate of the sprayed metal is set as 0.083 kg/s. Under the condition of constant melt temperature, gas injection angle, extension length of the guide pipe, and guide pipe diameter, the physical models of primary atomization, secondary atomization, and the crushing process are the volume of fluid multinomial flow/large-eddy model (LEM), the discrete phase model, and the Taylor analogy breakup model, respectively. The solver function uses the pressure-based transient method to simulate and calculate the atomization process of superalloy melts.

CFD adopts the discretized finite volume method, the calculation domain is a 2D plane model, and the atomized gas state is a constant flow incompressible state. Therefore, the flow control equation of the fluid is a plane incompressible constant current N-S equation:

(1) $$\left\{\begin{array}{c} \frac{\partial u_{i}}{\partial x_{i}}+\frac{\partial u_{j}}{\partial x_{j}}=0\\ \frac{\partial u_{i}}{\partial t}{+u}_{i}\frac{\partial u_{i}}{\partial x_{i}}{+u}_{j}\frac{\partial u_{i}}{\partial x_{j}}=-\frac{\partial \rho }{\partial x_{i}}+\frac{\mu }{LV}\left(\frac{\partial^{2}u_{i}}{\partial x_{i}^{2}}+\frac{\partial^{2}u_{i}}{\partial x_{j}^{2}}\right)\\ \frac{\partial u_{j}}{\partial t}{+u}_{i}\frac{\partial u_{j}}{\partial x_{i}}{+u}_{j}\frac{\partial u_{j}}{\partial x_{j}}=\frac{\partial \rho }{\partial x_{j}}+\frac{\mu }{LV}\left(\frac{\partial^{2}u_{j}}{\partial x_{i}^{2}}+\frac{\partial^{2}u_{j}}{\partial x_{j}^{2}}\right) \end{array}\right\}$$

where *u_i_* and *u_j_* are the velocity components in the directions of *x_i_* and *x_j_*, respectively; *ρ* is the fluid density; *V* is the characteristic flow velocity; *L* is the characteristic length; and *μ* is the fluid viscosity. In addition, the turbulence model for the interaction between gas and liquid metal flow in the atomization process mainly includes LEM, direct numerical simulation, and Reynolds average N-S model. The LEM can accurately solve the motion of turbulence at all turbulent scales, which can capture the turbulent transient process that are difficult to capture in the Reynolds stress model.

**Figure 1:**
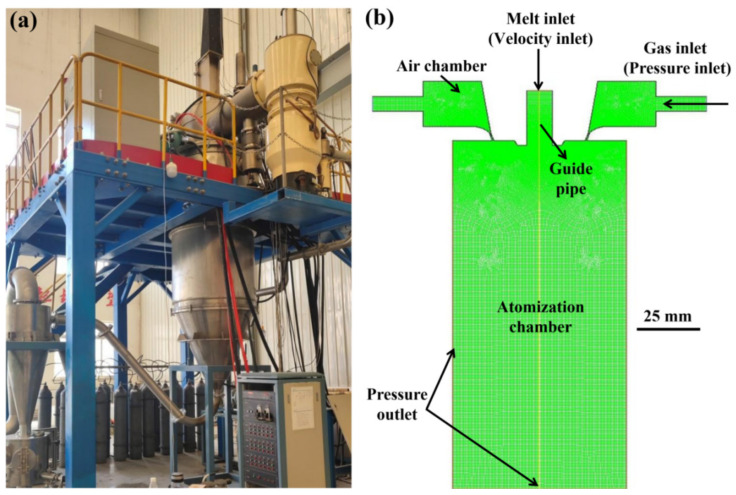
(**a**) Equipment illustration of the vacuum melting gas atomization pulverization. (**b**) Schematic illustration of the geometric model and meshing of the atomizing equipment.

This study adopts a self-developed VIGA pulverizing equipment (Figure 1a), which is composed of a melting chamber, an atomizer, an atomization chamber, a powder collection device, a supporting power supply system, a water cooling circulation system, a vacuum system, and a control system. Since the metal melt flows into the atomization chamber from the guide pipe to be subjected to complete atomization and the gas atomization equipment and atomizer are symmetrical, we simplify the three-dimensional structure with central symmetry into a two-dimensional plane axisymmetric structure. The obtained geometric model and the meshing scheme of the atomization equipment are illustrated in Figure 1b. Half of the two-dimensional axisymmetric geometric model of the atomization chamber is selected as the calculation area, the unstructured mesh option is adopted, and special mesh refinement is performed on the interaction area between the superalloy melt and the supersonic flow field in the guide pipe area. Argon is used as the atomization gas, the gas inlet is selected as the pressure inlet boundary, the superalloy melt inlet is used as the velocity inlet, and the left and lower boundaries of the nozzle are set as the pressure outlet.

## 3. Results and Discussion

### 3.1. Effect of Gas Pressure and Melt Flow Time on the Primary Atomization Process

**Figure 2:**
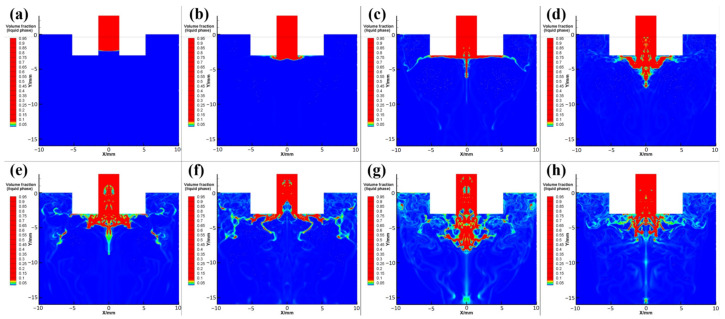
Flow pattern images of superalloy melt in the primary atomization process after of different flow times: (**a**) 4.0 ms, (**b**) 5.0 ms, (**c**) 6.0 ms, (**d**) 7.0 ms, (**e**) 8.0 ms, (**f**) 9.0 ms, (**g**) 10.0 ms, and (**h**) 11.0 ms.

Primary (initial) atomization is the process of stripping, breaking, and dispersing the columnar molten metal stream flowing from the guide pipe into a liquid film or large droplets [15,25,26]. Figure 2 shows the flow patterns of the superalloy melt in different stages of the atomization process, in which the extension length of the guide pipe, the guide pipe diameter, the injection angle, the atomization pressure, and the melt superheat are set to 3 mm, 3 mm, 36°, 5 MPa, and 200 K, respectively.

When the superalloy melt flows out of the liquid guide pipe under its own gravity and suction pressure, it first encounters the obstruction of the reverse airflow in the backflow area and cannot continue to flow downward. Thus, the melt flows radially to both sides along the direction of the airflow, forming a thin disk shape (Figure 2a–c). With further exposure to the airflow, the superalloy melt begins to break at the lower part and at the edge of its thin disk, and its flow pattern presents an umbrella shape (Figure 2d–f). At the edge of the umbrella structure, the superalloy melt is peeled off from the continuous melt flow and is spheroidized under the action of surface tension to form larger metal droplets, which then continue to break up in the turbulent layer (Figure 2g). At this time, the flow pattern of the superalloy melt appears like an inverted mushroom cloud. Because the melt and gas are injected continuously and stably during atomization and the process is in a dynamic equilibrium state, the crushing morphology of superalloy melt shows the dynamic alternating change in the form of umbrella shapes and inverted mushroom cloud shapes (Figure 2e–h).

**Figure 3:**
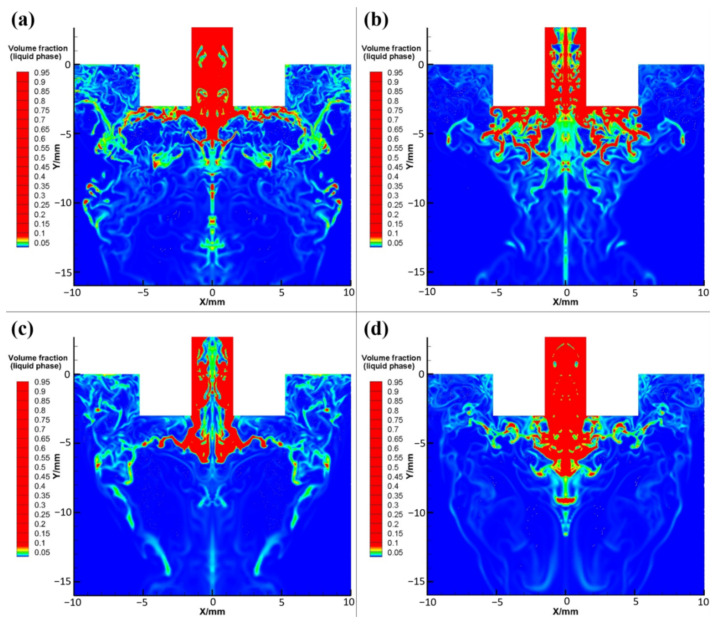
Flow pattern images of superalloy melt in the primary atomization process with different atomization pressures: (**a**) 2 Mpa, (**b**) 3 MPa, (**c**) 4 MPa, and (**d**) 5 MPa.

At the same time of the process, flow patterns of the primary atomized superalloy melt at different atomization pressures are shown in Figure 3, in which the red and blue parts represent the superalloy melt and atomization gas, respectively. As shown in Figure 3a–c, with the increase in atomization pressure, the amount of superalloy melt flowing into the atomization chamber gradually decreases, and the melt extends wider to both sides. As a result, a significant reduction in the proportion of large-size droplets and liquid film (red area) occurs after the melt column is stripped. It is worth noting that the increase in atomization pressure can increase the suction pressure at the bottom of the guide pipe, decrease the suction effect of gas on the melt, and decrease the amount of superalloy melt flowing into the atomization chamber at the same time, resulting in a gradual increase in the gas flow rate and gas velocity in the reflux area. Consequently, the gas–liquid mass flow ratio and relative gas–liquid velocity increase; thus, the atomization effect of gas on the melt is enhanced, and the droplets and the liquid film are more finely broken and dispersed after atomization. Furthermore, when the atomization pressure increases to 5 MPa, a greater amount of melts is forced to stick to the top inner wall of the atomization chamber near the outlet of the guide pipe and flow along the radial direction. At this time, the crushing strength of the gas on the liquid is weakened, resulting in insufficient crushing and the appearance of particles with large sizes, large flakes, or long fibrous powders (Figure 3d).

### 3.2. Effect of Gas Pressure on the Secondary Atomization Process

Secondary atomization refers to the process that the larger droplets or liquid film obtained from the superalloy melt after primary atomization are further broken into smaller droplets in the supersonic expansion zone of the airflow [27,28]. Generally, the droplet diameter after primary atomization is mostly greater than 0.4 mm, which is far from the standard of fine metal powder. Therefore, the primary atomization process is essentially preparation for the secondary atomization, and the crushing effect of the secondary atomization has the most direct impact on the final particle size of the powder.

**Figure 4:**
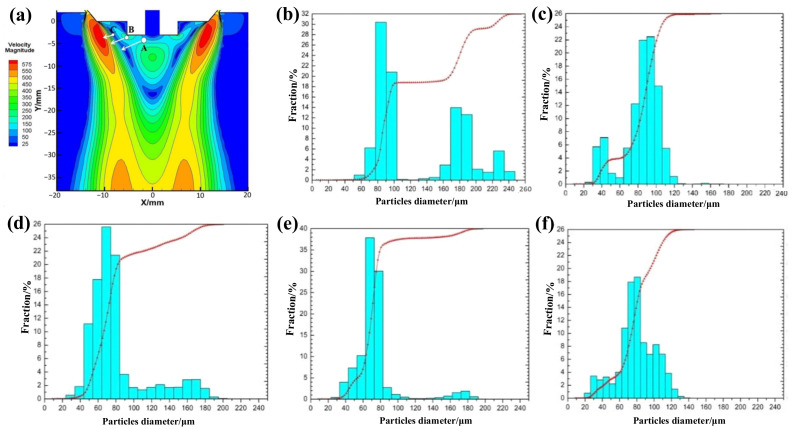
(**a**) Selection of injection source position at an atomization pressure of 5 MPa. (**b**–**f**) Particle size distribution at different atomization pressures: (**b**) 1 MPa, (**c**) 2 MPa, (**d**) 3 MPa, (**e**) 4 MPa, and (**f**) 5 MPa.

As shown in Figure 4a, the geometric model, melt properties, and grid structure are kept unchanged, and an injection mode composed of three injection streams is adopted. The diameters of the three initial droplets in each group are set to 0.7, 0.6, and 0.5 mm, respectively, the temperatures are set to 1863 K, and take A, B, C as the injection source. The particle tracer function in FLUENT software is used to capture the escaped particle information at the exit boundary of the model, and the particle size distribution of droplets after secondary atomization is statistically obtained at different atomization pressures, as shown in Figure 4b–f. At an atomization pressure of 1 MPa, the powder particle size distribution range is very wide, and the content of large-particle powder with a particle size above 160 μm is as high as 40% (Figure 4b). At an atomization pressure of 2 MPa, the powder particle size distribution range is significantly narrower than that at 1 MPa and is mainly distributed in the range of 80–100 μm, while almost no powder with a particle size above 160 μm is produced (Figure 4c). When the atomization pressure is 3 MPa, only a small amount (ca. 5%) of powder with particle sizes above 160 μm is generated, and the particle size distribution of the most abundant powder fraction is 40–180 μm (Figure 4d).

At an atomization pressure of 4 MPa, the particle size distribution range of the largest powder fraction is almost the same as that obtained at a pressure of 3 MPa, but the powder fraction with particle sizes above 120 μm is significantly reduced from 20% to 5%, and the average particle size is lower (Figure 4e). Furthermore, when the atomization pressure increases to 5 MPa, the distribution range of powder particle sizes becomes slightly widened, but the content of fine powder with particle sizes of 20–40 μm increases, while powder with particle sizes above 120 μm almost disappears, which greatly reduces the average particle size of the entire powder (Figure 4f). In short, with increasing atomization pressure, the range of powder particle size distribution gradually narrows, and the highest particle size peak in the powder particle size distribution histogram gradually shifts to smaller particle sizes, i.e., the average powder particle size gradually decreases.

**Figure 5:**
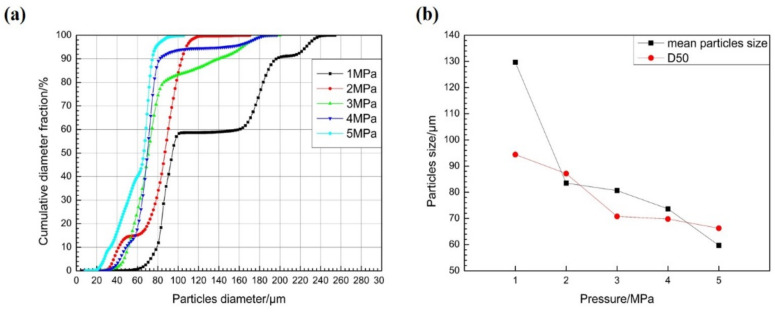
(**a**) Cumulative distribution curves of powder particle sizes at different atomization pressures. (**b**) Variation curves of average particle size and D50 value of powder with atomization pressures.

Next, Figure 5a shows the cumulative distribution curve of powder particle size obtained from secondary atomization statistics under different atomization pressure. When the atomization pressure gradually increases from 1 to 2, 3, 4, and 5 MPa; the cumulative distribution curve of powder particle size moves to the left correspondingly; and the content of large- and small-particle-size powder decreases and increases gradually, respectively. Moreover, the peak type of particle size distribution gradually changes from a wide multi-peak to a narrow single peak, and the single-peak shifts to the direction of smaller particle size. The powder median diameter (D50) decreased from 94.42 μm to 87.15, 70.80, 69.82, and 66.27 μm.

Figure 5b shows the variation of average particle sizes and D50 values of the powders obtained at different atomization pressures. When the atomization pressure gradually increases from 2 MPa to 3, 4, and 5 MPa, the D50 value of the powders decreases steadily, while the decrease in average particle size becomes increasingly larger. Importantly, the average powder particle sizes change by 2.4, 7.0, and 14.0 μm, respectively. Therefore, to obtain superalloy powder with fine particle sizes and a narrow distribution (i.e., improve the yield of fine powder) by VIGA, one could consider increasing the atomization gas pressure if the equipment safety and production costs allow.

**Figure 6:**
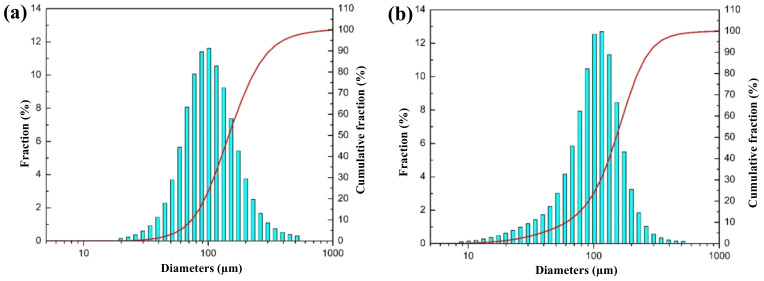

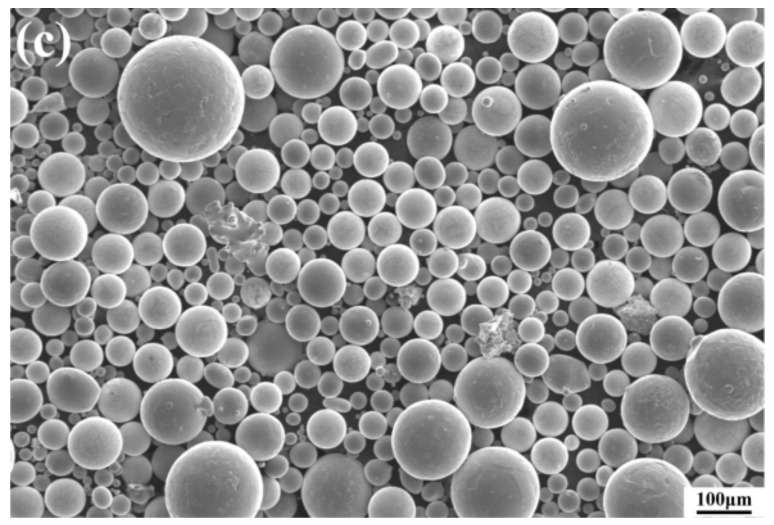
Particle size distribution and cumulative distribution curve of the actually prepared powder: (**a**) 80-mesh sieve powder and (**b**) 100-mesh sieve powder. (**c**) SEM image of powder prepared at an atomization pressure of 12 MPa.

To better guide the actual gas atomization production, we use the self-developed VIGA equipment to conduct a pulverizing experiment with an argon cylinder pressure of 12 MPa [15,29]. Generally, the particle size range of nickel-based superalloy powder for selective laser melting printing is under 53 μm. The particle sizes of the obtained powder after sieving with 80-mesh and 100-mesh sieves are measured with a laser particle size analyzer, respectively, and the particle size distributions and cumulative curves are shown in Figure 6. Compared with the numerical simulation results in Figure 4, the breaking of alloy droplets in the turbulent zone is similar to the actual pulverizing results, and the particle size distribution range and cumulative distribution curve are very close. Moreover, Figure 6c shows that the surface of nickel-based superalloy powders prepared by gas atomization is a smooth surface and has good sphericity, and its particle size distribution is also basically consistent with Figure 4. These results indicate that the crushing effect of gas atomization with a constant supply pressure of 12 MPa and 5 MPa cylinders is similar, and the main secondary atomization occurs in the boundary turbulent layer between the airflow expansion zone and the backflow zone.

## 4. Conclusions

In summary, we use the CFD method to numerically simulate the effect of different gas pressures on the particle properties of nickel-based superalloy powders prepared by VIGA. The results show that in the process of atomization, the crushing morphology of the superalloy melt is an alternate transformation of umbrella and inverted mushroom cloud shapes. The interaction of gas–liquid two-phase flow leads to a disorder of the entire flow field structure, which increases with the increase in atomization pressure, thereby promoting the atomization of the alloy melt. In the process of secondary atomization, the particle size distribution of alloy powder becomes narrower with the increase in atomization pressure, and the median diameter and average particle size decrease. When the atomization pressure increases from 2 to 5 MPa, the average particle size of the alloy powder decreases by 2.4, 7.0, and 14.0 μm, respectively. In addition, the powder particle size distribution obtained in the actual production process at an atomization pressure of 12 MPa is in good agreement with that of the alloy powder obtained by numerical simulation (5 MPa). This study can not only provide technical support for the optimization of the VIGA process for the preparation of superalloy powder, but also reduce production cost while ensuring powder quality.

## Data Availability

Not applicable.
